# Reads Binning Improves the Assembly of Viral Genome Sequences From Metagenomic Samples

**DOI:** 10.3389/fmicb.2021.664560

**Published:** 2021-05-21

**Authors:** Kai Song

**Affiliations:** School of Mathematics and Statistics, Qingdao University, Qingdao China

**Keywords:** metagenome, Markov chain, virus, assembly, contigs

## Abstract

Metagenomes can be considered as mixtures of viral, bacterial, and other eukaryotic DNA sequences. Mining viral sequences from metagenomes could shed insight into virus–host relationships and expand viral databases. Current alignment-based methods are unsuitable for identifying viral sequences from metagenome sequences because most assembled metagenomic contigs are short and possess few or no predicted genes, and most metagenomic viral genes are dissimilar to known viral genes. In this study, I developed a Markov model-based method, VirMC, to identify viral sequences from metagenomic data. VirMC uses Markov chains to model sequence signatures and construct a scoring model using a likelihood test to distinguish viral and bacterial sequences. Compared with the other two state-of-the-art viral sequence-prediction methods, VirFinder and PPR-Meta, my proposed method outperformed VirFinder and had similar performance with PPR-Meta for short contigs with length less than 400 bp. VirMC outperformed VirFinder and PPR-Meta for identifying viral sequences in contaminated metagenomic samples with eukaryotic sequences. VirMC showed better performance in assembling viral-genome sequences from metagenomic data (based on filtering potential bacterial reads). Applying VirMC to human gut metagenomes from healthy subjects and patients with type-2 diabetes (T2D) revealed that viral contigs could help classify healthy and diseased statuses. This alignment-free method complements gene-based alignment approaches and will significantly improve the precision of viral sequence identification.

## Introduction

Viruses are obligate intracellular parasites that probably infect all cellular forms of life (Breitbart and Rohwer, 2005). At least 10^31^ virus particles exist globally at any given time in most environments in which the number of detectable virus particles exceeds the number of bacterial cells by 10-fold (Edwards and Rohwer, 2005; Rosario and Breitbart, 2011; Mokili et al., 2012; Chow and Suttle, 2015). Bacterial viruses represent the most numerous viral entities, and they affect host bacteria (Breitbart and Rohwer, 2005). For example, in the human gut microbiome, alterations in the relative abundances of gut viruses can influence type-2 diabetes (T2D) and inflammatory bowel disease (Norman et al., 2015; Ma et al., 2018). In soil and aquatic environments, phages can also play important roles in influencing bacterial biogeochemical processes (Wommack and Colwell, 2000; Suttle, 2005; Kimura et al., 2008).

Traditional views regarding virus–host interactions for viral communities have been limited due to virus-isolation techniques; in particular, a small fraction of viruses (less than 15%) could be isolated from known phyla of prokaryotic hosts (Roux et al., 2015b). However, high-throughput sequencing and metagenomic approaches have radically changed the state of virology research, with many more viruses now known solely based on sequence data than have been characterized experimentally (Labonte and Suttle, 2013; Dayaram et al., 2015, 2016; Rosario et al., 2015; Krupovic et al., 2016). Many metagenomic studies rely on the approach of selectively capturing and sequencing viral particles outside prokaryotic host cells; however, sequencing cellular fraction samples can also reveal viral sequences. Previous research showed that the human gut prokaryote metagenome was comprised of 4–17% viral sequences (Minot et al., 2011). Phage sequences found in the prokaryotic host cell could be from lysogenic virus that integrated into the host genome, or the lytic virus bound to specific host cells not released to the surrounding environment (Knowles et al., 2016; Song, 2020). Thus, existing virome metagenomic studies cannot capture sequences from viruses replicating in prokaryotic host cells.

Metagenome sequences can be viewed as a mixture of viral, bacterial, and other eukaryotic sequences. Mining viral sequences from metagenomes can increase the understanding of viruses and their associations with host cells. The first crucial step is to identify viral sequences from metagenomes. Tools for identifying proviruses from bacterial genomes have been developed previously, including Phage_Finder (Fouts, 2006), Prophinder (Lima-Mendez et al., 2008), PHAST (Zhou et al., 2011), and PhiSpy (Akhter et al., 2012). These prophage detectors generally use sliding windows and a reference-based search for known virus genes, and then predict those regions as being derived from proviruses. These tools are not suitable for identifying viral sequences from metagenome sequences because most assembled metagenome contigs are short and possess few or no predicted genes; furthermore, most virus genes in metagenomes are not similar to known virus genes. For example, it is estimated that only about 15% of viruses in the human gut microbiome and 10% in the ocean are similar to known viruses (Hurwitz and Sullivan, 2013; Norman et al., 2015).

Previous studies performed to identify viral sequences from metagenomic samples mainly used *de novo* assembly first, after which viral contigs were predicted from the assembled contigs, using alignment-based (Roux et al., 2015a) and alignment-free methods (Ren et al., 2017, 2020; Fang et al., 2019). However, assembling metagenomic sequence reads to generate viral and bacterial contigs may produce many errors that are caused by the mosaic organization of viral genomes or sequence similarities between viral and bacterial genomes (Hendrix et al., 1999). Thus, it is necessary to obtain sequencing reads from viral genomes before assembly to reduce errors.

*K*-mer-based sequence comparisons have been widely used in many areas, including phylogenetic tree construction (Song et al., 2013), metagenomic sample comparison (Jiang et al., 2012; Song et al., 2019), metagenomic reads binning (Lu et al., 2017), virus classification (Song, 2020), and identifying motifs (Narlikar et al., 2013). VirFinder (Ren et al., 2017) is a *k*-mer-based machine-learning method that avoids gene-based similarity searches. Thus, the advantage of this method is that it can detect viral contigs as short as 1,000 bp. In contrast, metagenomic assemblies produce contigs of various lengths ranging from hundreds of base pairs (bp) to 10^5^ bp or more. The VirFinder tool requires the construction of training models for contigs of different length ranges. Otherwise, it cannot achieve the best performance in terms of viral-sequence detection. In addition, the read length from metagenomic samples is mainly 200–300 bp, which is beyond the accuracy range of the VirFinder method. PPR-Meta (Fang et al., 2019) is also a *k*-mer-based method combined with deep learning. Like VirFinder, PPR-Meta relies on different modules for predicting sequences of different lengths, but had superior performance. Therefore, it is necessary to develop a new method to classify short reads before *de novo* assembly that do not rely on the length of contigs used for training.

In this study, I developed a Markov model-based method, VirMC, to identify viral sequences from metagenomic data. VirMC uses Markov chains to model the sequence signatures and construct a scoring model using a likelihood test to distinguish viral and bacterial sequences. I evaluated the performance of VirMC in detecting viral sequences (including novel viruses) over a range of read lengths, including short reads (200 or 300 bp) up to contig-level reads (≥1,000 bp). VirMC showed better or similar performance with VirFinder and PPR-Meta in identifying short and long reads. Also, VirMC exhibited improved performance over VirFinder and PPR-Meta in correctly identifying viral sequences from contaminated metagenomic samples with eukaryotic sequences. VirMC was applied to classify sequencing reads before *de novo* assembly, which improved the assembly of viral genomic sequences from metagenomic samples. VirMC was also used to identify viral sequences in human gut-metagenomic data from healthy subjects and patients with T2D. Some of these viral contigs could be used to predict the disease status, demonstrating the potential use of viral sequences in diagnosing human diseased states. The software is available at https://github.com/songkai1987/VirMC.

## Materials and Methods

### Viral and Bacterial Genomes Databases

In this study, I used databases that were also used previously by Ren et al. (2017). The databases were constructed by downloading 1,562 viral genomes that infected Bacteria and Archaea, and the 31,986 prokaryote genomes (including Bacteria and Archaea) from the National Center for Biotechnology Information (NCBI) before 31 May 2015. I collected another 753 viral genomes and 5,865 bacterial genomes from the NCBI after May 2015. To mimic fragmented metagenomic sequences, for a given length (*L*) (*L* = 200, 300, 400, 500, 1,000, or 3,000 bp), viral genomes were split into non-overlapping fragments of length *L*, and the same number of non-overlapping fragments of length *L* were randomly subsampled from the bacterial genomes. Fragments generated for viral genomes discovered between 1 January 2014 and 31 May 2015 were used as validation sets, and those generated after 1 June 2015 were used as testing sets. To generate validation and testing datasets containing 10, 50, or 90% viral contigs, the number of viral contigs was set as shown in Table 1, and the contigs were combined with nine times more, equal numbers, or ninefold less randomly sampled bacterial contigs discovered after 1 June 2015, respectively. These datasets were named as Simulated Data Set One which were used to evaluate the performance of VirMC in classifying the viral and bacterial contigs.

**TABLE 1:** The number of fragments generated from viral and bacterial genomes discovered after 1 January 2014.

	**Number of viral fragments**	
**Fragment length**	**January 2014–May 2015**	**After May 2015**
200 bp	125,666	266,204
300 bp	83,832	177,330
400 bp	62,833	132,890
500 bp	50,350	106,228
1,000 bp	25,087	52,902
3,000 bp	8,246	17,345

	**Number of bacterial fragments (90%)**	
**Fragment length**	**January 2014–May 2015**	**After May 2015**

200 bp	1,130,994	2,395,836
300 bp	754,488	1,595,970
400 bp	565,497	1,196,010
500 bp	453,150	956,052
1,000 bp	225,783	476,118
3,000 bp	74,214	156,105

	**Number of bacterial fragments (10%)**	
**Fragment length**	**January 2014–May 2015**	**After May 2015**

200 bp	13,963	29,579
300 bp	9,315	19,704
400 bp	6,982	14,766
500 bp	5,595	11,804
1,000 bp	2,788	5,878
3,000 bp	917	1,928

*For testing datasets with 50% viral contigs, the number of bacterial contigs was the same as the number of viral contigs. For testing datasets with 10 and 90% viral contigs, the number of bacterial contigs was shown in this table.*

### Human, Fungi, and Protozoan Genomes Databases

The human genome was downloaded from ensemble databased^1^. In addition, I downloaded the 277 Fungi genomes and 83 Protozoan genomes that were also used previously (Ponsero and Hurwitz, 2019). To mimic fragmented metagenomic sequences, the genomes were also split into non-overlapping fragments of length *L* (*L* = 200, 300, 400, 500, 1,000, or 3,000 bp). To generate the metagenomic samples with contaminated eukaryotic sequences, the contigs were randomly sampled from Human, Fungi, and Protozoan genomes to combine with viral and bacterial contigs in testing dataset, respectively. These datasets were named as Simulated Data Set Two which were used to evaluate the performance of VirMC in classifying identifying viral contigs in simulated contaminated metagenomic samples.

### Building Markov Models From Viral and Bacterial Genome Sequences

For a set or a single genomic sequence *S*, *N*(*w*) is the total number of occurrences of the *k*-mer *w* = *w*_1_*w*_2_…*w*_*k*_ and its complementary *k*-mer $\bar{w}$, *w*_*i*_ ∈ 𝒜 = {*A*,*C*,*G*,*T*},*i* = 1,2,…,*k*. The *k*-th-order Markov chain is defined using the 4^*k*^×4 transition probability matrix. The maximum-likelihood estimation of the Markov chain’s conditional probability of observing nucleotides *w*_*k+1*_ given preceding nucleotides *w*_1_*w*_2_…*w*_*k*_ is

$$P_{M}\,\left(w_{k+1}|w_{1}\,w_{2}\,\ldots \,w_{k}\right)=\frac{N\,\left(w_{1}\,w_{2}\,\ldots \,w_{k+1}\right)}{N\,\left(w_{1}\,w_{2}\,\ldots \,w_{k}\right)}$$

### The Markov Model-Based Prediction Method

First, the *k*-th order Markov chains were used to model viral and bacterial genomic sequences obtained before 1 January 2014. I calculated the GC frequency of each bacterial genomic sequence, grouped these bacterial genomic sequences into different bins using the quantiles of the GC-frequency distribution, and then constructed Markov models using the genomic sequences in each bin.

Suppose the *M* different *k*-th order Markov chains ($M_{k}^{1,\text{virus}},M_{k}^{2,\text{virus}},\ldots ,M_{k}^{M,\text{virus}}$) were used to model the virus genomic sequences before 1 January 2014, so was well with bacterial genomic sequences. For a contig sequence *y* = *y*_1_*y*_2_…*y*_*N*_, the log-likelihood under the Markov chain *M*_*k*_ is

$$\text{LL}\,\left(y|M_{k}\right)=\frac{1}{N-k}\,\sum_{i=1}^{N-k}l\,o\,g\,P_{M_{k}}\,\left(y_{i+k}|y_{1}\,y_{2}\,\ldots \,y_{i+k-1}\right)$$

Then, the statistic λ was defined to measure the likelihood that the contig belonged to a virus or bacteria:

(1) $$\lambda =\frac{\text{max}\left(\text{LL}\left(y|M_{k}^{1,\text{virus}}\right),\text{LL}\left(y|M_{k}^{2,\text{virus}}\right),..,\text{LL}\left(y|M_{k}^{M,\text{virus}}\right)\right)}{\text{max}\left(\text{LL}\left(y|M_{k}^{1,\text{host}}\right),\text{LL}\left(y|M_{k}^{2,\text{host}}\right),..,\text{LL}\left(y|M_{k}^{M,\text{host}}\right)\right)}$$

If λ > 1, then the probability of a contig belonging to virus was larger than it belonging to bacteria. If λ < 1, then the probability of a contig belonging to bacteria was larger than it belonging to a virus.

In real metagenomic experiments, the assembled contigs or sequencing reads have various lengths. To compare the scores of contigs with different lengths, for each query contig, a *p* value was computed by comparing the score with the null distribution, that is, the distribution of scores for the tested bacterial contigs. The *p* value was computed as the fraction of tested bacterial contigs that had greater scores than the score of the query sequence.

### Analysis of Simulated Metagenomic Samples

Metagenomic samples were simulated based on species-abundance profiles derived from a real human gut metagenomic sample (accession ID SRR061166, Platform: Illumina) from the HMP (Peterson et al., 2009), which is commonly used for metagenomic data analysis (Boisvert et al., 2012; Luo et al., 2014; Brittnacher et al., 2016; Rampelli et al., 2016). These datasets were named as Simulated Data Set Three.

I used the abundance profiles generated by a previous study with VirFinder (Ren et al., 2017) using 1,562 complete virus genome sequences and 2,698 complete bacterial genome sequences downloaded from NCBI RefSeq. Then, I used NeSSM software (Jia et al., 2013) to simulate metagenomic samples with paired-end short reads (150 bp in length) in an Illumina MiSeq setting mode, based on the abundance profiles.

Samples with 20 and 40 million sequencing reads were generated using the viral and bacterial genomic sequences. The relative abundances of viral and bacterial reads were kept the same, and then the virus and bacterium reads were mixed to make 20 and 50% viral samples. The λ score of each paired-end read was calculated. The paired-end reads with scores higher than a threshold value were predicted to be from viral genomes and were used for assembly. metaSPAdes software (Bankevich et al., 2012; Nurk et al., 2017) was used for *de novo* assembly of the simulated metagenome samples, using the command “spades.py–meta.” Only contigs of ≥300 bp were used for downstream analysis.

To obtain the true labels for the assembled contigs, reads in the simulated data were mapped to the set of contigs using “bwa mem.” A contig was labeled as a viral contig if it was assembled from reads generated by viral genomes. Similarly, a contig was labeled as a bacterial contig if it was assembled from reads generated by bacterial genomes. A contig was labeled as chimeric if it was assembled from a mixture of viral and bacterial reads. The assembled viral contigs were mapped to the viral genomes to estimate the assembly precision between filtered and unfiltered assemblies. The genome completeness of the assembled contigs was evaluated using the software CheckV (Nayfach et al., 2020).

### Assembly and Analysis of Human Gut Metagenomic Samples From a T2D Study

Human gut metagenomic samples from T2D patients and healthy controls in China were downloaded from NCBI Sequence Read Archive under accession numbers SRA045646 and SRA050230 (Qin et al., 2012). Forty samples were selected randomly as the “training set” and were comprised of 20 samples from patients with T2D and 20 samples from healthy controls. Another 40 samples were randomly selected and used as the “testing set.”

The λ score of each paired-end read in the training dataset was calculated. The reads with the top 10% values were filtered for assembly. metaSPAdes software (Bankevich et al., 2012; Nurk et al., 2017) was used to cross-assemble the filtered reads in the training dataset using the default setting.

COCACOLA (Lu et al., 2017) was used to cluster viral contigs predicted by VirMC, based on tetranucleotide frequencies and contig coverages. The fragments per kilobase per million mapped reads (FPKM) values were determined by mapping sample reads with Bowtie2 software (Langmead and Salzberg, 2012), using the default settings, and were averaged for each bin. FPKM values were used to train a classification model to classify the disease status (0 for healthy subjects and 1 for patients with T2D). A logistic-regression model with lasso regularization was used to enhance the prediction accuracy and interpretability. Thus, a subset of viral bins was chosen to achieve the best prediction accuracy. A ROC curve was used to evaluate the classification performance with another 40 samples in the testing dataset.

## Results

Viral and bacterial genomic sequences could be modeled as different Markov chains, based on their varied GC frequency and *k*-mer-usage patterns. The guanine-cytosine (GC) frequency of each bacterial genomic sequence was calculated. These bacterial genomic sequences were grouped into different bins using the quantiles of the GC-frequency distribution, and a Markov model was constructed for each bin.

### The Effects of the Number of Cluster Bins and the Order of Markov Models

In this part, the Simulated Data Set One was used to determine how the number of cluster bins and the order of the Markov model would affect the predictive performance. To generates a prediction for each query sequence, VirMC first extracted the *k*-mer features from the bacterial and viral genomic sequence before 1 January 2014 and then generated a λ score (query score) based on the Markov models, with a higher score indicating a higher possibility that the sequence was viral.

**FIGURE 1:**
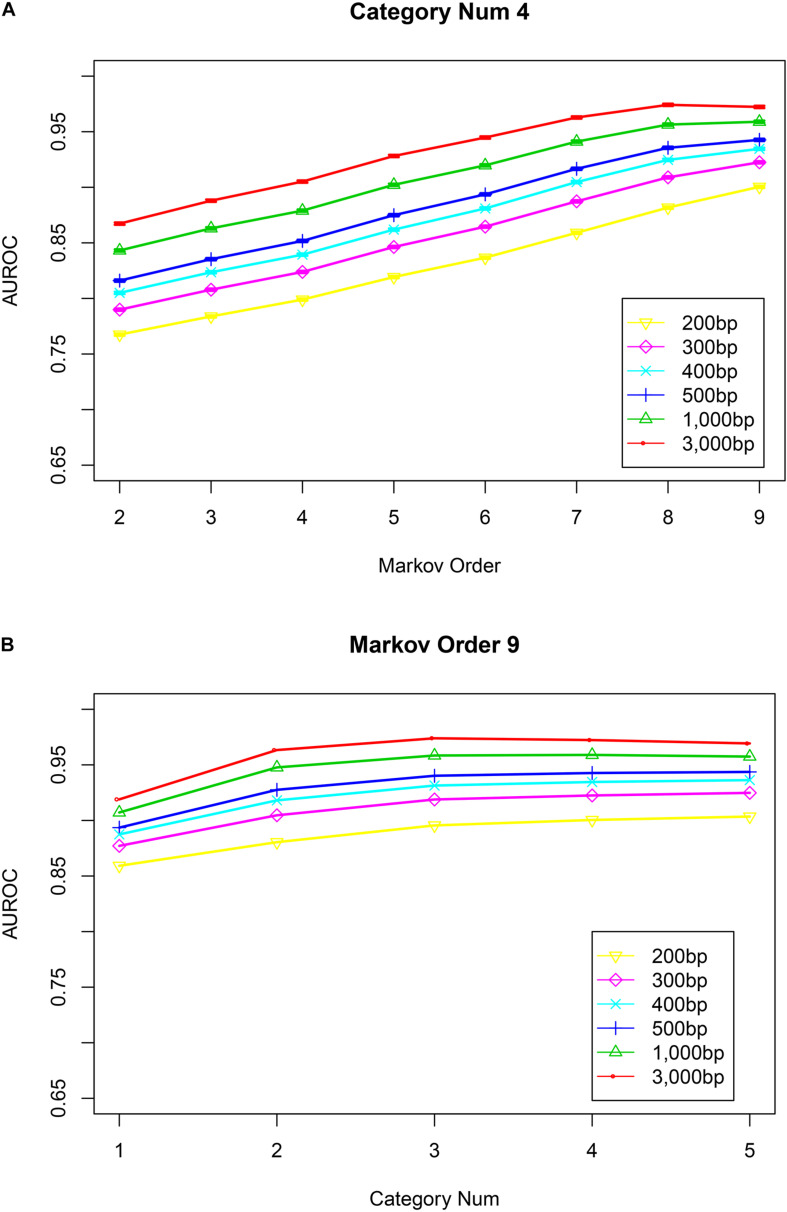
The impact of Markov orders and numbers of cluster bins on the performance of VirMC. Error bars depict the standard error determined from 30 bootstrap samples from the validation dataset. **(A)** Area under the curve for receiver operator characteristic (AUROC) curves are shown when VirMC was trained using different Markov orders and tested using different contig lengths. **(B)** AUROC curve values for VirMC results when the training model used a Markov order of 9 and different numbers of cluster bins.

After training the Markov model, VirMC was validated with equal numbers of viral and bacterial contigs subsampled from genomes sequenced between 1 January 2014 and 31 May 2015; these genome sequences were also used in a previous study (Ren et al., 2017). Receiver operating characteristic (ROC) curves were used to validate the performance of this classifier. The area under the curve (AUC) values increased as the Markov order and contig length increased (Figure 1A, number of bins = 4; Supplementary Figure 1, number of bins = 1, 2, 3, and 5). For contigs with a length of 3,000 bp, performance was relatively stable at a Markov order of ≥8. For contigs with a length of ≤1,000 bp, the performance still appeared to increase above a Markov order of 8. Even for contigs with lengths of 300 or 400 bp, the AUC values were >0.90 when the Markov order was ≥8. For contigs with a length of 200 bp, the AUC value was almost 0.90 when the Markov order was 9. Thus, these high area under the curve for receiver operator characteristic (AUROC) curve scores demonstrated that my method could correctly identify viral sequences with high-throughput sequencing reads.

Next, I determined the relationship between the classification performance and the number of cluster bins. For contigs with a length of 3,000 bp, the AUC values slightly decreased when the number of cluster bins was larger than 4 (Figure 1B, Markov order = 9). For other contigs with lengths ranging from 200 to 1,000 bp, the AUC values were relatively stable when the number of cluster bins was larger than 4. For other fixed Markov orders (ranging from 5 to 8), the performance was also relatively stable at cluster bin number of ≥4 (Supplementary Figure 2). Markov models constructed from 3, 4, or 5 bins showed similar performances.

**FIGURE 2:**
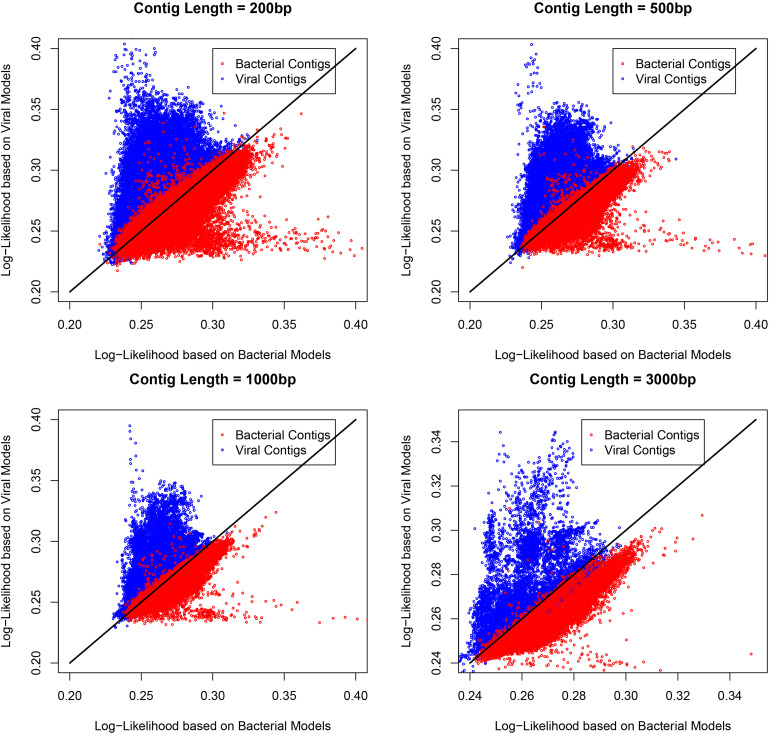
The scatterplots of the log-likelihoods for contigs with different length. The number of cluster bins was 4 and Markov order was 9 for constructing the training model. The length of contigs were 200, 500, 1,000, and 3,000 bp.

Metagenomic datasets may contain different proportions of viral and bacterial contigs, which can potentially affect the performance of a tool that is validated based on equal proportions of viral contigs. In practice, the fraction of viral contigs will vary with different types of samples, so the VirMC was evaluated as described above using subsampled viral and bacterial contigs, sequenced between 1 January 2014 and 31 May 2015, but with mixtures containing 10 and 90% viral sequences. The AUROC scores did not differ significantly between the mixtures with different fractions of viral sequences (Supplementary Figure 3). Therefore, I fixed the number of cluster bins at 4 and the Markov order at 9 for subsequent analyses. The scatterplots of the log-likelihoods for contigs with different length under cluster bin number of 4 and Markov order of 9 for these contigs were shown in Figure 2.

### Comparing the Performances of the VirMC, VirFinder, and PPR-Meta Tools

I compared the ability of VirMC to correctly identify viral contigs with the previously developed methods, PPR-Meta (Fang et al., 2019) and the VirFinder method (Ren et al., 2017). To provide a fair comparison, the training and validating sets of contigs subsampled from viral and bacterial genomes sequenced before 31 May 2015 in Simulated Data Sets One were combined and used for the training datasets. The contigs obtained from viral and bacterial genomes sequenced after 1 June 2015 with different mixture fractions were used for the testing datasets.

**FIGURE 3:**
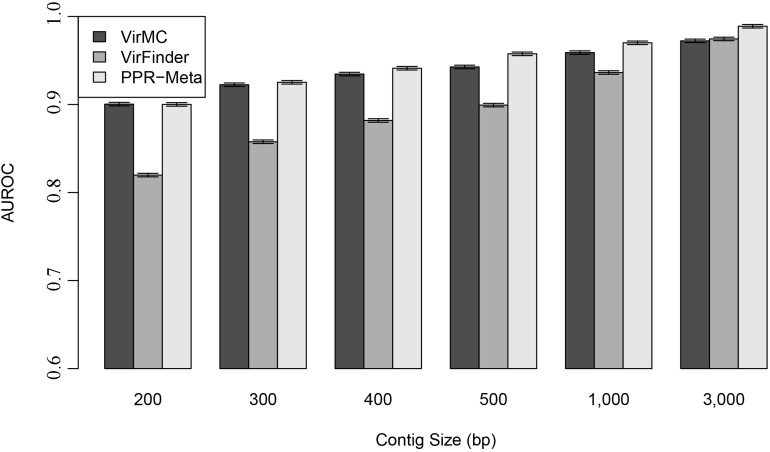
Performance of VirMC, VirFinder, and PPR-Meta in predicting viruses for contigs with different lengths. The error bars depict standard errors determined from 30 bootstrap samples from the testing dataset. AUROC are shown when VirMC was trained using a Markov order of 9 and a cluster bin number of 4.

Area under ROC curves were used to evaluate the performance of these three classifiers. For contigs with length larger than 500 bp, PPR-Meta had larger AUROC values than VirMC and VirFinder, suggesting that PPR-Meta performed better than VirMC and VirFinder when the contig length was long (Figure 3). However, for contigs with length less than 400 bp, VirMC had similar performances with PPR-Meta. VirMC and PPR-Meta all performed better than VirFinder for contigs length less than 1,000 bp. The AUROC scores also showed no obvious difference between the equal and unequal fractions of viral mixtures (Supplementary Figure 4).

### Comparing the Performances of VirMC, VirFinder, and PPR-Meta Tools on Contaminated Metagenomic Samples

In addition to viral and bacterial genomic sequences, metagenomic samples also contain eukaryotic sequences, such as Human, Fungi, and Protozoa. In this part, the Simulated Data Set Two was used to evaluate the performance of different tools on contaminated metagenomic samples.

**FIGURE 4:**
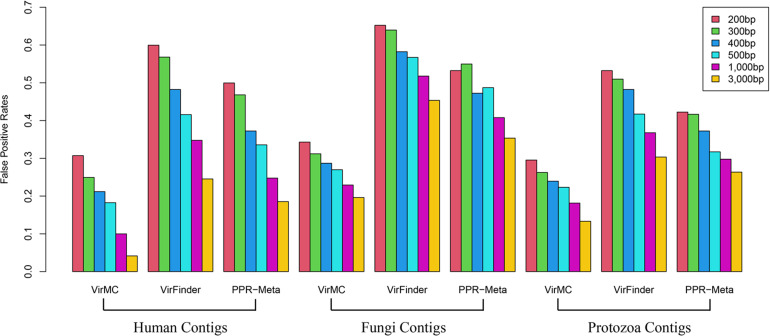
The False Positive Rates of VirMC, VirFinder, and PPR-Meta for classifying eukaryotic contigs from Human, Fungi, and Protozoan. The eukaryotic genomes were divided into fragments of different lengths and then used for evaluation.

Firstly, I tested the classification accuracy of these three tools for eukaryotic sequences. VirFinder and PPR-Meta had a much stronger misclassification of eukaryotic sequences than VirMC (Figure 4). For contigs from human genomes with a length of 200 bp, the False Positive Rates (FPRs) of VirFinder and PPR were both more than 50%, while the FPR of VirMC was about 30%. For contigs with a length of 3,000 bp, the FPR of PPR-Meta was about 20%, while the FPR of VirMC was only 4%. VirMC also had better performance in classification of eukaryotic sequences from Fungi and Protozoa (Figure 4).

**FIGURE 5:**
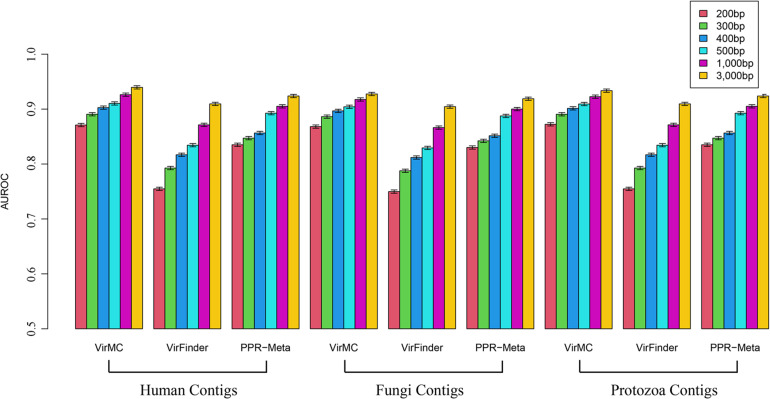
The AUROC values of VirMC, VirFinder, and PPR-Meta in predicting viral contigs from contaminated metagenomic samples with Human, Fungi, and Protozoan sequences. The contigs were randomly sampled from Human, Fungi, and Protozoan genomes to combine with viral and bacterial contigs in testing datasets, respectively.

In order to evaluate the abilities of these three tools to identify viral sequences from contaminated metagenomic samples, the contigs were randomly sampled from Human, Fungi and Protozoan genomes to combine with viral and bacterial contigs, respectively. The eukaryotic sequences were chosen to account for 10% of the entire dataset. VirMC performed better than VirFinder and PPR-Meta, especially for contigs length lower than 400 bp (Figure 5). For samples with human contigs, the AUROC values of VirMC were 0.871, 0.891, 0.903, 0.910, 0.926, and 0.940 for 200-, 300-, 400-, 500-, 1, 000-, and 3,000-bps contigs, which were larger than the values of VirFinder and PPR-Meta, respectively. For metagenomic samples with Fungi or Protozoa contigs, VirMC also performed better than VirFinder and PPR-Meta.

### The Abilities of VirMC to Predict Novel Viruses

To assess the ability of VirMC to identify novel viruses, I focused on the 45 viruses used in a previous study conducted by Ren et al. (2017) that showed no significant nucleotide similarity (blastn searches, *E* values < 10^–5^) to previously deposited viral genome sequences, which could be considered novel viruses. Using a *p* value cut-off of 0.01, VirMC predicted 38 viruses, while VirFinder only predicted 30 viruses (Supplementary Table 1).

### Application: Assembling Viral Contigs From Simulated Metagenomic Samples Using VirMC

**FIGURE 6:**
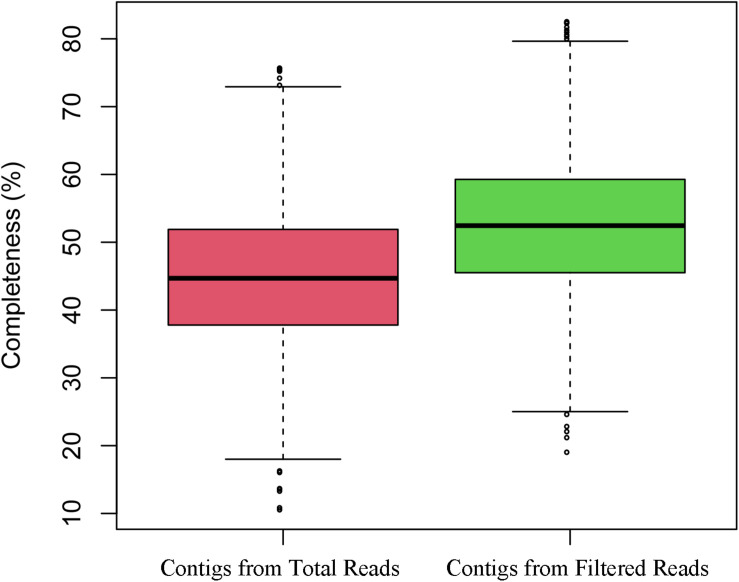
The completeness of the assembled contigs with filtered reads and with total reads.

Because VirMC showed better performance in classifying viral sequences with relatively short lengths (200 and 300 bp), I evaluated the accuracy and total length of viral contigs assembled from simulated metagenomic samples in Simulated Data Set Three by filtering the potential non-viral paired-end sequences. A simulated human gut metagenome with 40 million reads (20 million host reads and 20 million viral reads) was generated using NeSSM software (Jia et al., 2013) by subsampling reads from viral and bacterial reference genomes found in a real gut metagenomic sample (Human Microbiome Project; HMP) at their respective relative abundances (Ren et al., 2017). The log-likelihood score of each paired-end read was calculated using VirMC. The reads with scores lower than a cut off value of 1.0026 (Supplementary Figure 5, False Discovery Rate ≤ 0.9 and Call Rate ≥ 0.8 for viral reads) were filtered before assembling. Assembly was performed separately with filtered and unfiltered reads, using metaSPAdes software (Bankevich et al., 2012; Nurk et al., 2017). Contigs with lengths of ≥300 bp were reserved for subsequent analysis, and each contig was definitively assigned as bacterial, viral, or ambiguously chimeric (see the “Materials and Methods” section). The assembly precision was defined using the number of correctly assembled viral contigs divided by the number of viral and ambiguously chimeric contigs. Assembly with filtered reads produced more viral contigs and higher precision than assembly with unfiltered reads (Table 2). For contigs with lengths of 3,000–5,000, 5,000–10,000, and ≥10,000 bp, the precision rates were as high as 89.3, 91.2, and 88.1%, respectively, for assemblies with filtered reads. However, the precision rates were only 80.5, 79.8, and 61.7%, respectively, when performing assemblies with unfiltered reads. Because the genome completeness of short contigs was very low, I evaluated the completeness of the assembled contigs longer than 5 kb using the software CheckV (Nayfach et al., 2020). The assembled contigs with filtered reads were more complete than the contigs with total reads (Figure 6). The total length of viral contigs assembled with filtered reads was 47.9 Mb, which was 23% higher than the viral contigs assembled with total reads (39.0 Mb). The coverages of viral genomes were 61.4 and 50.0% for viral contigs assembled from filtered reads and total reads, respectively. Because the short contigs (<300 bp) were filtered in the analysis and the sequencing depth was not deep enough, the coverages of viral genomes were not very high. To study the effects of the sequencing depth and the fraction of viral sequences in each sample, simulated metagenomic samples were generated for 20 and 40 million total reads using different viral and bacterial proportions (20% viral reads and 80% bacterial reads; see the Materials and Methods section). The performances in terms of the assembled length and precision were better with filtered reads than those obtained with raw unfiltered reads, for the depth of 20 and 40 million simulated reads, indicating that variation in the sequence depth and composition ratio did not affect the assembly results (Supplementary Table 2).

**TABLE 2:** Comparison of assembly precision, using filtered reads and total reads.

	**20 Million reads**				**40 Million reads**			
	**Filtered reads**		**Total reads**		**Filtered reads**		**Total reads**	
**Contig length**	**Contig num**	**Precision**	**Contig num**	**Precision**	**Contig num**	**Precision**	**Contig num**	**Precision**
300–500 bp	11,622	0.969	8,339	0.947	9,496	0.959	4,457	0.937
500–1,000 bp	6,740	0.948	6,251	0.918	5,817	0.931	4,157	0.908
1,000–2,000 bp	5,131	0.928	4,605	0.891	5,183	0.901	3,590	0.877
2,000–3,000 bp	1,719	0.929	1,596	0.858	2,030	0.878	1,504	0.843
3,000–5,000 bp	1,359	0.917	1,173	0.833	1,532	0.893	1,250	0.805
5,000–10,000 bp	865	0.935	716	0.812	1,071	0.912	878	0.798
>10,000 bp	697	0.900	628	0.631	828	0.881	886	0.617
Assembly length	44.7 Mb		37.4 Mb		47.9 Mb		39.0 Mb	
N50	6.8 kb		5.2 kb		7.2 kb		5.6 kb	
Coverage	57.3%		47.9%		61.4%		50.0%	

### Application: Identification and Analysis of Viral Communities in Human Gut Metagenomes From a T2D Study

Type-2 diabetes is a heterogeneous and multifactorial disease, influenced by several different genetic and environmental factors, which has become a major public health issue worldwide. Qin et al. (2012) previously reported that gut microbiota was linked to and contributed to T2D, based on deep, next-generation shotgun sequencing of DNA extracted from stool samples from Chinese patients with T2D and non-diabetic control subjects. Here, I used VirMC to reanalyse the dataset generated by Qin et al. to identify viruses in these metagenomes.

The λ score of each paired-end read from 20 healthy subjects and 20 patients with T2D (generated from 242 Gb of total sequence data) was calculated. The paired-end reads with the top 10% values (λ score ≥ 1.016) were filtered for assembly purposes. Only the resulting 15,390 contigs with high quality (>80% completeness) and longer than 1,000 bp in length were retained in order to achieve high prediction accuracy. VirMC predicted 6,134 contigs as viral sequences, respectively. The False Positive Rates were estimated at 5% using *q* values, which were estimated based on the False Discovery Rate method (Storey, 2003; Dabney et al., 2010).

Contigs were binned using the COCACOLA framework (Lu et al., 2017), based on *k*-mer frequencies and abundance patterns across samples, in order to group similar contigs. This produced 153 bins for contigs predicted by VirMC. The abundance profiles of the contig bins across 40 samples from healthy subjects and patients with T2D were used to train classification models and distinguish the health status. A logistic regression model with lasso regularization was used to enhance the prediction accuracy and interpretability. These models were then tested on another 40 samples from the same study using AUROC scores. The binned contigs had AUROC score of 0.74 which showed that they could be used to classify disease states.

## Discussion

In this study, I developed a Markov model-based method to identify viral sequences from metagenomic data. The VirMC method uses Markov chains to model sequence signatures and construct a scoring model, using a likelihood test to distinguish viral sequences from bacterial sequences. One of the biggest contributions of VirMC is that it can correctly identify viral sequences as short as 200 or 300 bp. In recent years, high-throughput sequencing technology has been applied to an increasing number of metagenomic studies and has produced massive amounts of data (Qin et al., 2010, 2012; Falony et al., 2016). Assembly of these sequencing data is very time consuming and requires large computer memory resources; moreover, assembling metagenomes poses great and complex challenges due to genetic diversity, DNA repeats, and DNA transfer between strains (Miller et al., 2010). Therefore, identifying viral sequences from assembled metagenomic contigs is difficult and shows low precision, unless it is a prophage. In this work, I developed another approach to identify viral contigs from metagenomic data through two steps. First, I used VirMC to filter the paired-end sequencing reads to obtain the potential viral reads, and second, I assembled the filtered reads to obtain the viral contigs. The assembled length and precision of the viral contigs were both improved using this approach.

Viral sequences identification based on Markov Chain (VirMC) works by training Markov models on known viral and bacterial genomic sequences to predict the likelihood of *k*-mer transition patterns used by viruses or bacterium. Compared with VirFinder and PPR-Meta, the advantage of VirMC is that it is not necessary to train different models for sequences with different lengths. VirMC is suitable and flexible for analysing metagenomic assembly results containing contigs of continuous lengths. Genomes are complex, hierarchically organized entities shaped largely by the forces of evolution. The primary sequence of a genome reflects both short- and long-range correlations, which can be viewed and modeled as Markov chains (Dehnert et al., 2005; Ren et al., 2016). Viruses and their prokaryotic hosts are very different biological entities, so different evolutionary pressures have shaped their genomic spatial scale correlations. Thus, the assumption of VirMC is that viruses and bacterial genomes can be modeled using different Markov chains.

To distinguish viral sequences from bacterial sequences, a statistic score, λ, which was based on likelihood ratio test was proposed in VirMC. The larger value of λ, the greater probability that the contig was from viral genomes. In order to illustrate how to choose the appropriate λ values in application, the relationship between Precision, Recall and λ values under different contig length was given (Supplementary Figure 6). For contigs with a length of ≤500 bp, when λ = 0.987, the Precision and Recall curves had an intersection which could be a cut-off value. For contigs with length of 1,000 and 3,000 bp, the λ value was 0.984. However, for the above λ values, the Precision and Recall values were all not very high (≤90%). To get viral contigs with high precision, the λ value should be increased. For contigs with a length of 200 bp and Precision larger than 0.95, the λ value should be larger than 1.013. For contigs with length of 1,000 and 3,000 bp, the λ value should be larger than 0.995 and 0.991, respectively.

Using a time point as the dividing criterion, VirMC was trained with the viral and bacterial genomic sequences obtained before May 2015 and correctly predicted the viral sequences found after June 2015, which demonstrates that my approach can be applied for classifying viral sequences in future metagenomics studies. It is estimated that only about 15% of viruses in the human gut microbiome and 10% in the ocean are similar to known viruses (Hurwitz and Sullivan, 2013; Norman et al., 2015). High-throughput sequencing technology has greatly changed viral research, with many more viruses now known solely from sequence data than have been characterized experimentally (Simmonds et al., 2017). The assembled contigs from metagenomic data are mostly short and contain little or no genes; therefore, reference-based virus prediction is difficult to be applied in this research field.

VirMC provides a new alignment-free *k*-mer-based approach for identifying viral sequences in metagenomic data. In a side-by-side comparison with VirFinder and PPR-Meta, PPR-Meta had better performance than VirMC, however, for shorter contigs (i.e., 200–400 bp), VirMC had similar performance with PPR-Meta. In addition to viral and bacterial sequences, metagenomic samples also contained other eukaryotic sequences which could affect the classification accuracy of these tools. When considering the contamination of eukaryotic sequences, VirMC had superior performance compared with VirFinder and PPR-Meta. VirMC provides another approach to improve the assembly of viral genomes from metagenomic data. This approach is to first identify viral reads and then assemble on them, which both improves assembly accuracy and length.

## Data Availability Statement

The original contributions presented in the study are included in the article/Supplementary Material, further inquiries can be directed to the corresponding author.

## Author Contributions

KS conceived of the project, developed the methods, performed the computations, and contributed to the final manuscript.

## Conflict of Interest

The author declares that the research was conducted in the absence of any commercial or financial relationships that could be construed as a potential conflict of interest.

## Footnotes

1 http://ftp.ensembl.org/pub/release-104/fasta/homo_sapiens/dna/Homo_sapiens.GRCh38.dna.alt.fa.gz
