# Supplementary material for: Reads Binning Improves the Assembly of Viral Genome Sequences From Metagenomic Samples
Source: Front Microbiol. 2021 May 21;12:664560. doi: 10.3389/fmicb.2021.664560 (PMC8175635; doi:10.3389/fmicb.2021.664560)
Supplement: Supplementary file 1 [file Data_Sheet_1.docx]

**Reads binning improves the assembly of viral genome sequences from metagenomic samples Supplemental Material**

Kai Song ^a,*^

^a^ School of Mathematics and Statistics, Qingdao University, Qingdao 266071, Shandong, China

^*^Corresponding author. Email: [songkai1987@126.com](mailto:songkai1987@126.com) (K. Song)

Figure S1: The impact of Markov orders on the performance of VirMC under different category number. Error bars depict standard error determined from 30 bootstrap samples from the testing dataset. Area under the curve for receiver operator curves (AUROC) are shown when VirMC was trained using different Markov orders and tested using different contig length under fixed category number 1, 2, 3 and 5.


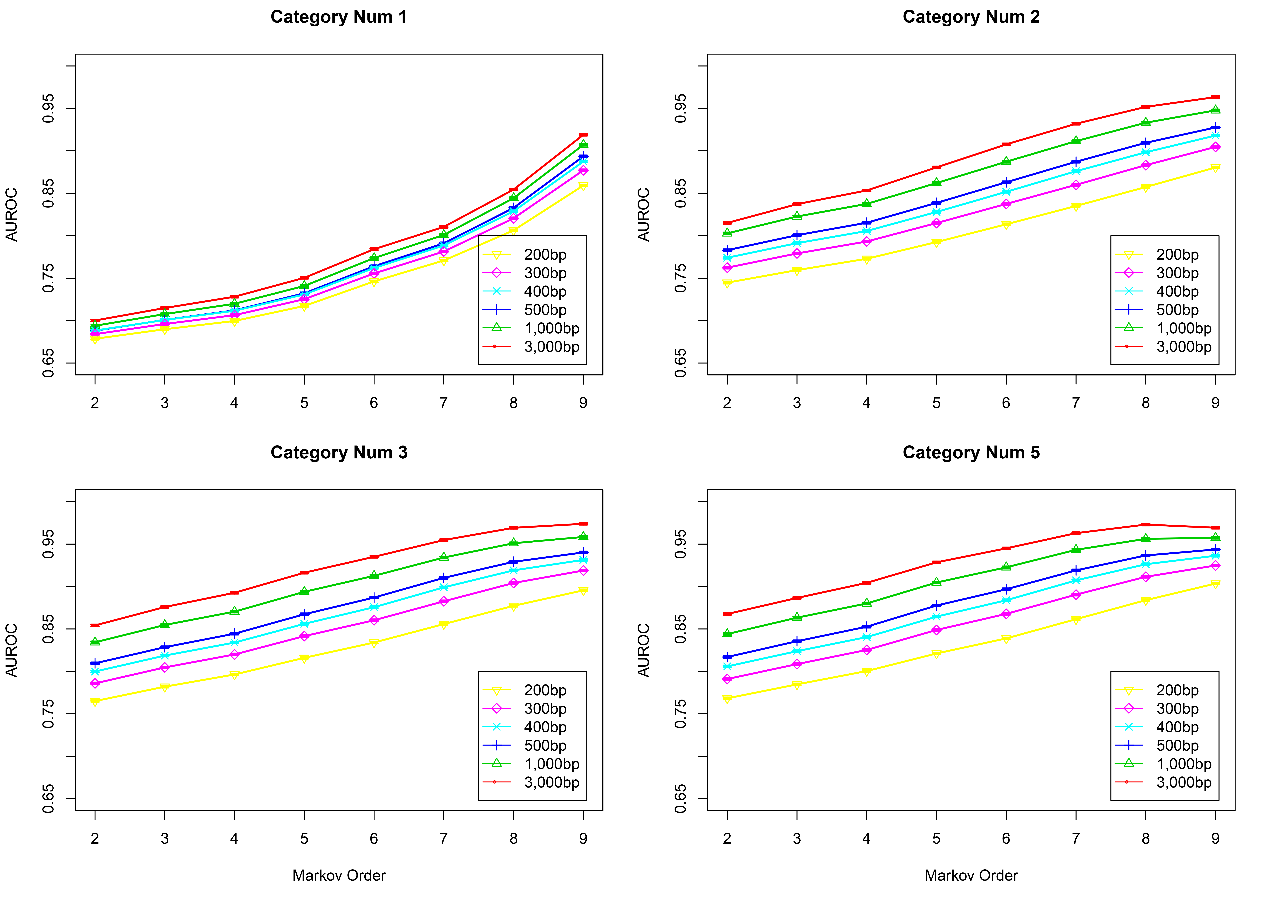


Figure S2: The impact of category number on the performance of VirMC under different Markov orders. Error bars depict standard error determined from 30 bootstrap samples from the testing dataset. AUROC values for VirMC results when training model using Markov order 5, 6, 7 and 8 and different category number.


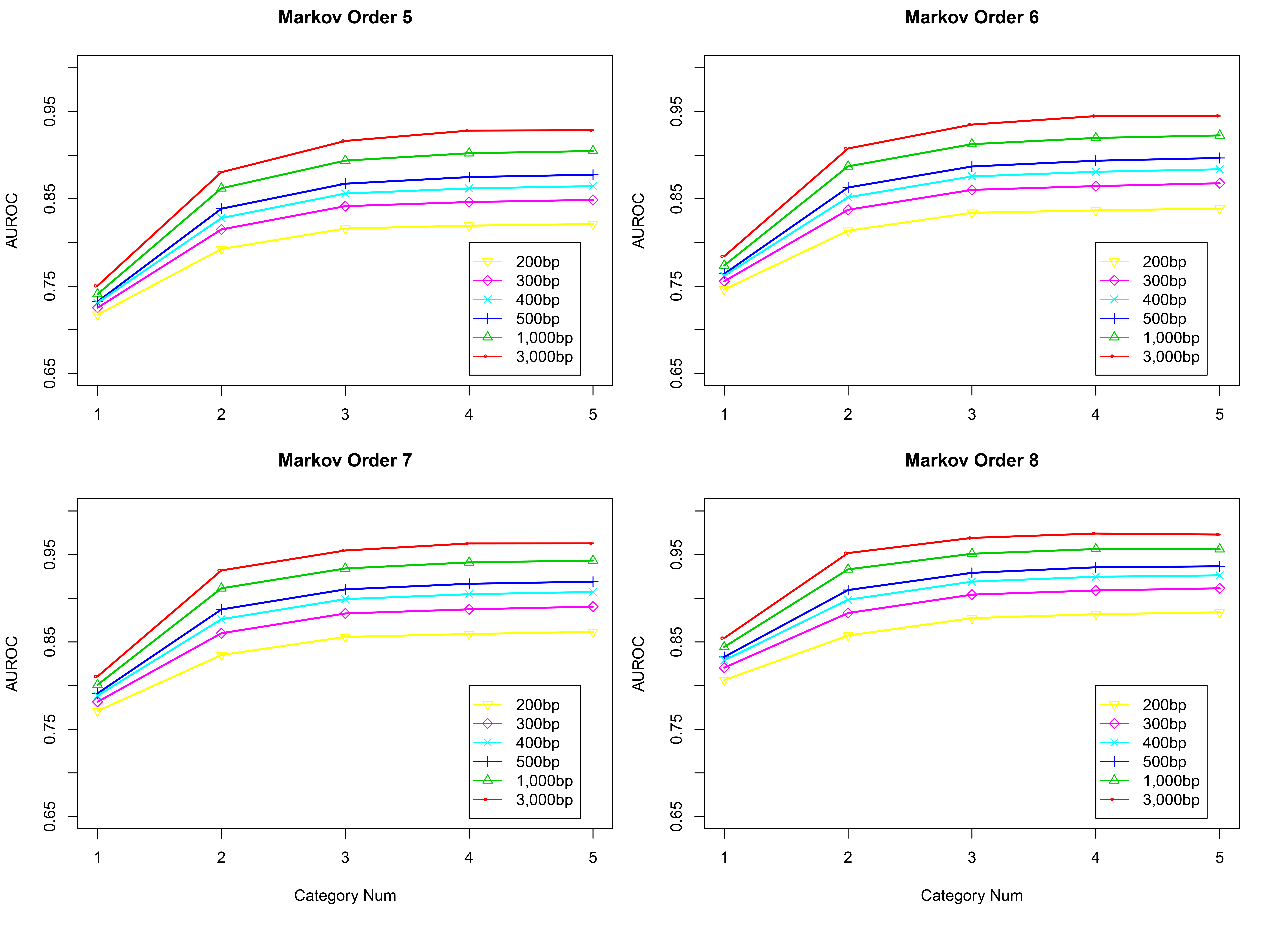


Figure S3: The impact of Markov orders on the performance of VirMC under different virus mixture fraction. Error bars depict standard error determined from 30 bootstrap samples from the testing dataset. Area under the curve for receiver operator curves (AUROC) are shown when VirMC was trained using different Markov orders and tested using different contig length under category number 4.


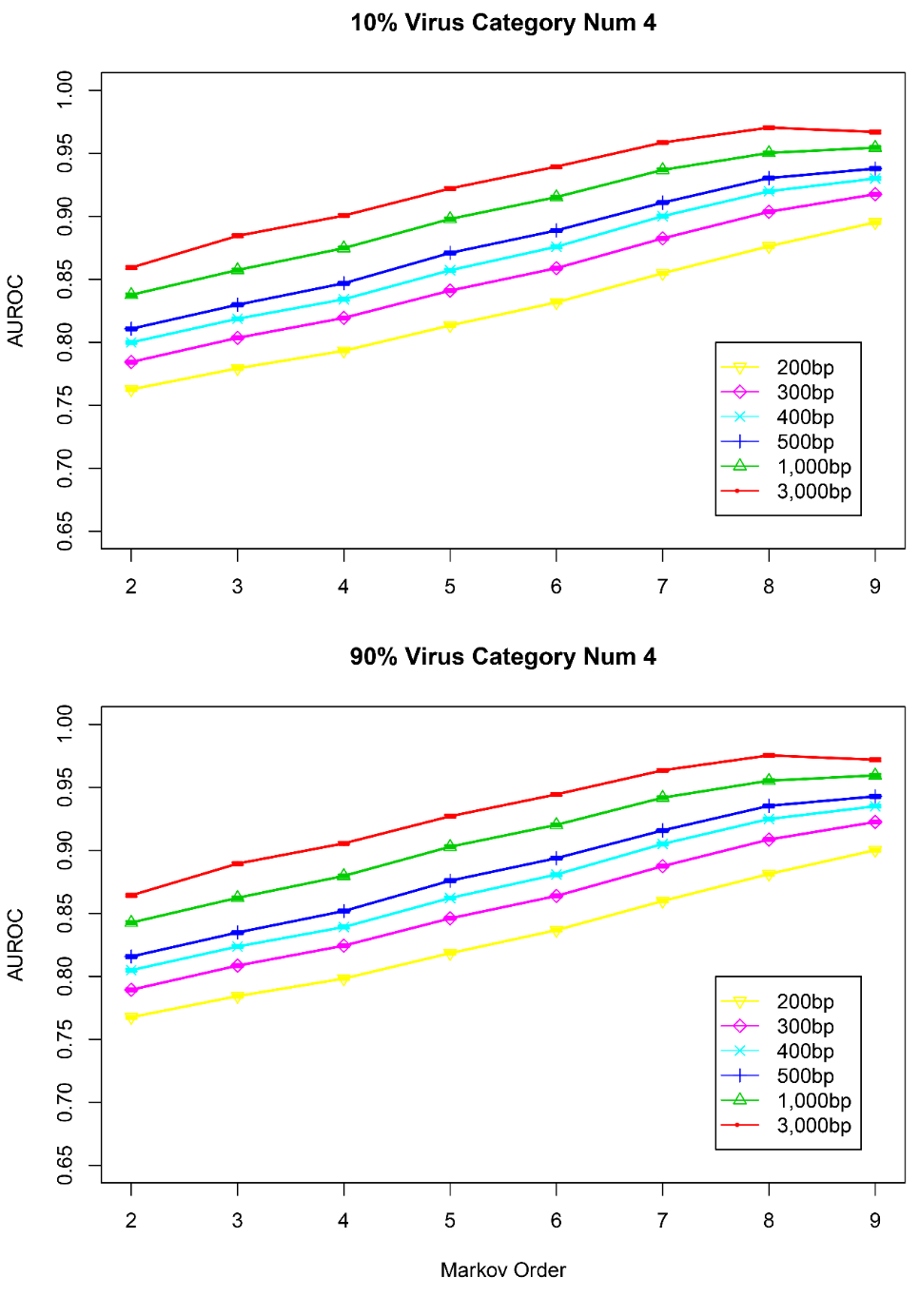


Figure S4: Viral prediction performance of VirMC, VirFinder and PPR-Meta for different contig length under different virus mixture fraction. Error bars depict standard error determined from 30 bootstrap samples from the testing dataset. Area under the curve for receiver operator curves (AUROC) is shown when VirMC was trained using Markov order 9 and category number 4.


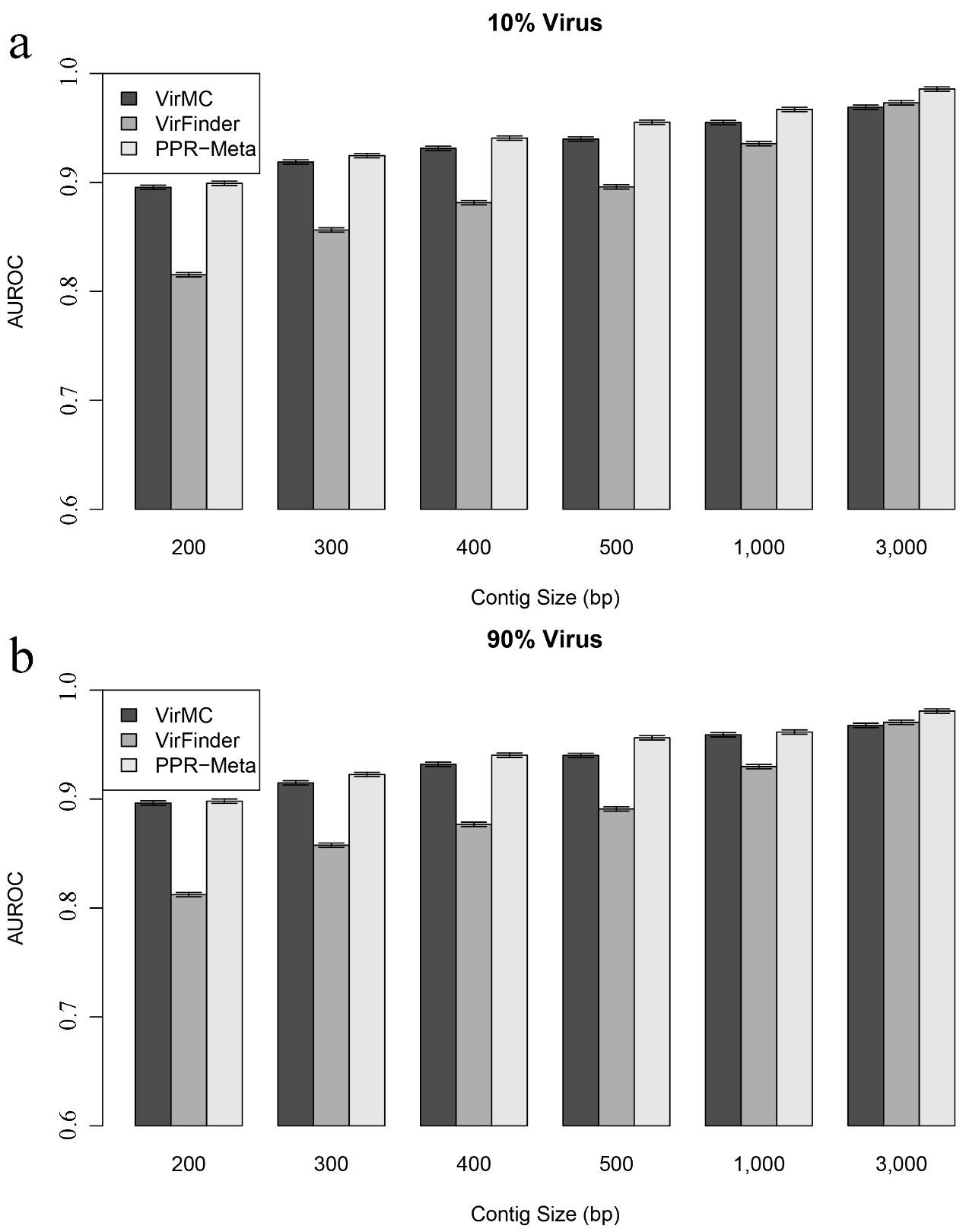


Figure S5: The λ score of NeSSM generate pair-end reads from host and virus genomic sequences.


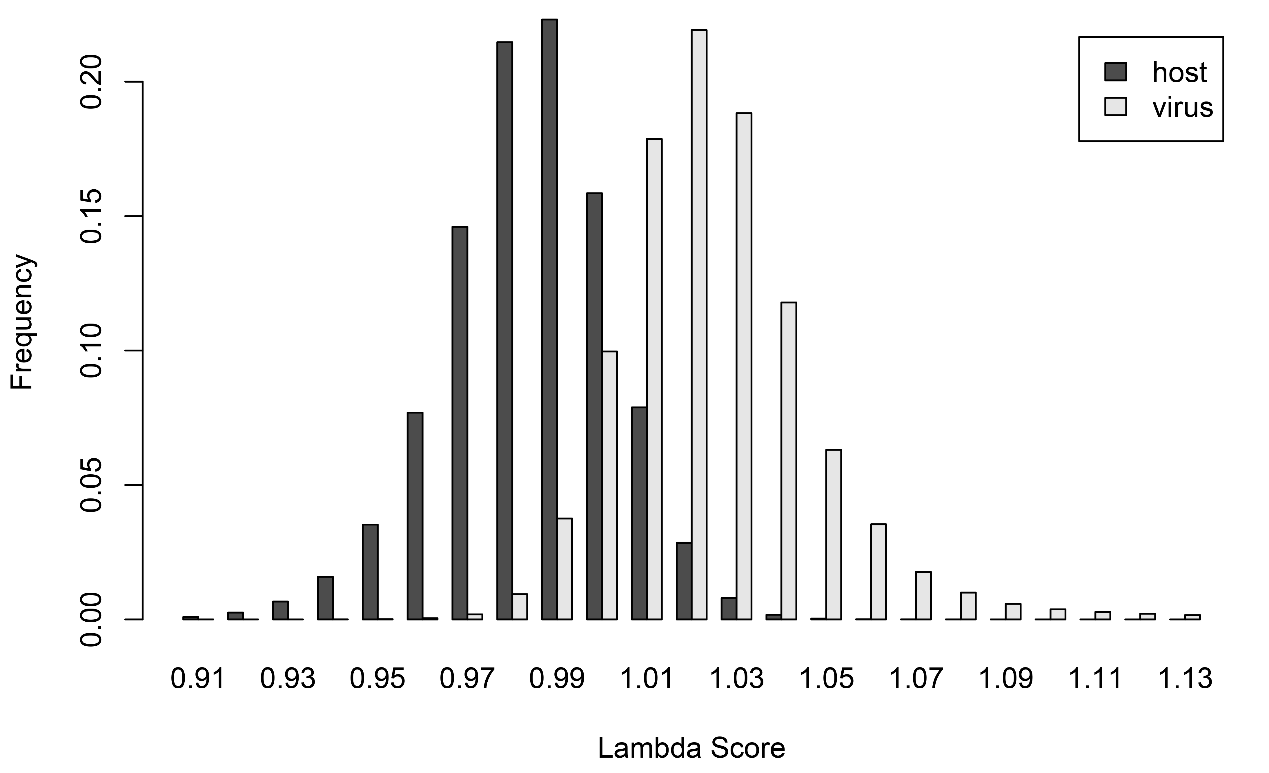


Figure S6. The relationship between Precision, Recall and λ values for contigs with different length.


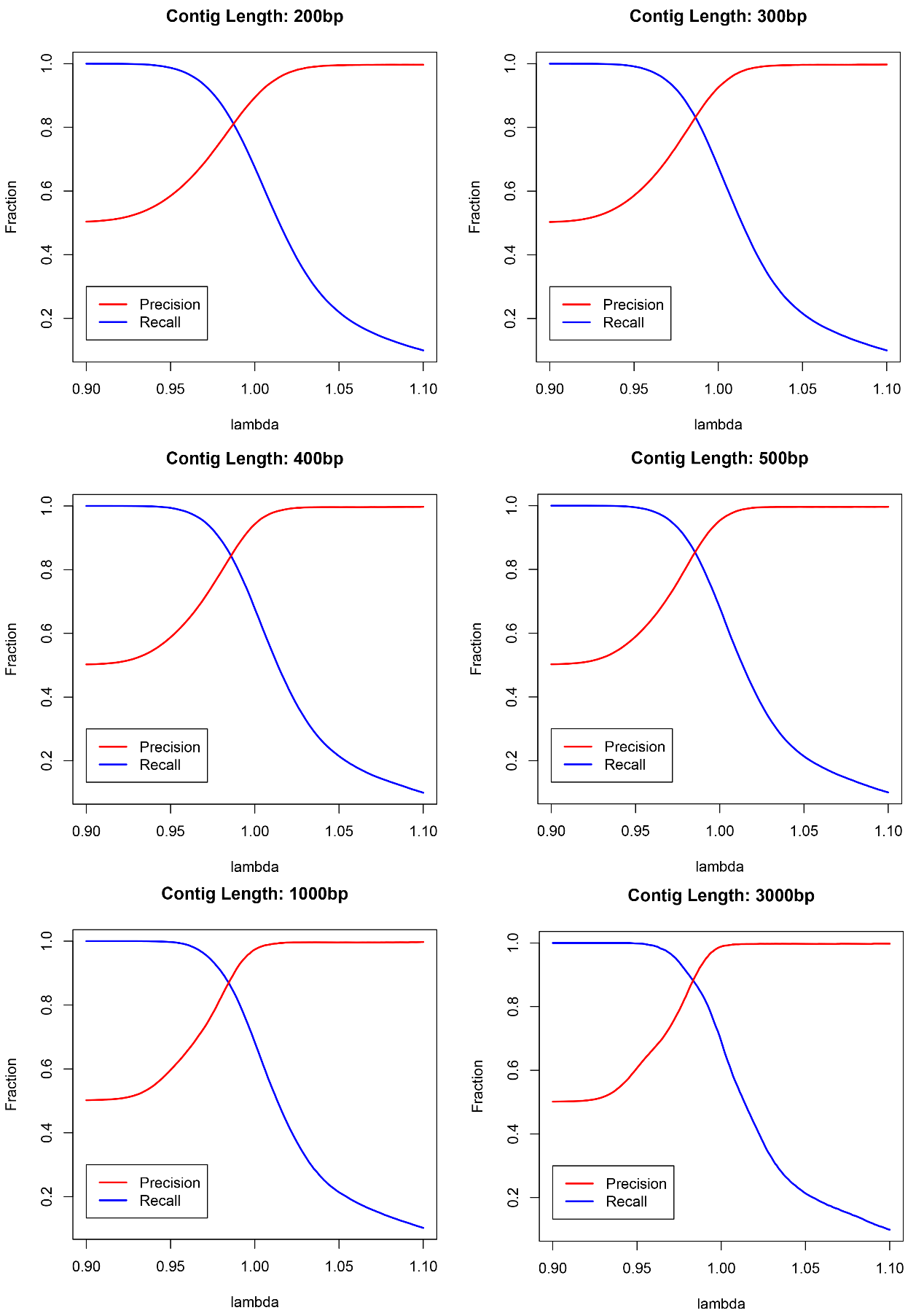


Table S1. VirMC prediction results for the 45 novel viruses that had no significant nucleotide similarity (blastn search, E value <10^–5^) to previously viral genome sequences.

| Virus name | NCBI accession | VirMC score | VirMC pvalue | VirMC results |
| --- | --- | --- | --- | --- |
| Oenococcus phage phi9805 | NC_023559 | 1.0051 | 0.0042 | TRUE |
| Oenococcus phage phiS11 | NC_023571 | 1.0053 | 0.0040 | TRUE |
| Oenococcus phage phiS13 | NC_023560 | 1.0077 | 0.0030 | TRUE |
| Eel River basin pequenovirus | NC_026665 | 1.0106 | 0.0023 | TRUE |
| Mycobacterium phage Adler | NC_023591 | 0.9842 | 0.1087 | FALSE |
| Vibrio phage X29 | NC_024369 | 0.9965 | 0.0175 | FALSE |
| Uncultured phage WW-nAnB strain 3 | NC_026613 | 1.0164 | 0.0015 | TRUE |
| Shewanella sp. phage 1/41 | NC_025458 | 1.0000 | 0.0094 | TRUE |
| Rhizobium phage vB_RglS_P106B | NC_023566 | 1.0011 | 0.0078 | TRUE |
| Psychrobacter phage Psymv2 | NC_023734 | 0.9981 | 0.0132 | FALSE |
| Erwinia phage Ea35-70 | NC_023557 | 1.0126 | 0.0019 | TRUE |
| Arthrobacter phage vB_ArtM-ArV1 | NC_026606 | 0.9903 | 0.0489 | FALSE |
| Vibrio phage VpKK5 | NC_026610 | 1.0236 | 0.0012 | TRUE |
| Microviridae IME-16 | NC_026013 | 0.9980 | 0.0134 | FALSE |
| Croceibacter phage P2559Y | NC_023614 | 1.0007 | 0.0083 | TRUE |
| Lactoccocus phage WP-2 | NC_024149 | 1.0133 | 0.0018 | TRUE |
| Aeromonas phage pAh6-C | NC_025459 | 1.0356 | 0.0009 | TRUE |
| Rhizobium phage vB_RleS_L338C | NC_023502 | 0.9945 | 0.0254 | FALSE |
| Rhodococcus phage ReqiPoco6 | NC_023694 | 1.0442 | 0.0007 | TRUE |
| Erwinia phage PhiEaH1 | NC_023610 | 1.0215 | 0.0013 | TRUE |
| Uncultured phage WW-nAnB strain 2 | NC_026612 | 1.0179 | 0.0014 | TRUE |
| Rhodococcus phage ReqiPepy6 | NC_023735 | 1.0450 | 0.0007 | TRUE |
| Idiomarinaceae phage Phi1M2-2 | NC_025471 | 1.0175 | 0.0015 | TRUE |
| Shewanella sp. phage 3/49 | NC_025466 | 1.0035 | 0.0053 | TRUE |
| Idiomarinaceae phage 1N2-2 | NC_025439 | 1.0160 | 0.0016 | TRUE |
| Clavibacter phage CN1A | NC_023549 | 1.0014 | 0.0073 | TRUE |
| uncultured phage WW-nAnB | NC_026582 | 1.0130 | 0.0019 | TRUE |
| Ruegeria phage DSS3-P1 | NC_025428 | 0.9943 | 0.0258 | FALSE |
| Vibrio phage CHOED | NC_023863 | 1.0209 | 0.0013 | TRUE |
| Shewanella sp. phage 1/44 | NC_025463 | 1.0097 | 0.0024 | TRUE |
| Mesorhizobium phagevB_MloP_Lo5R7ANS | NC_025431 | 1.0011 | 0.0079 | TRUE |
| Shewanella phage Spp001 | NC_023594 | 1.0159 | 0.0016 | TRUE |
| Enterococcus phage vB_Efae230P-4 | NC_025467 | 1.0151 | 0.0016 | TRUE |
| uncultured phage crAssphage | NC_024711 | 1.0188 | 0.0014 | TRUE |
| Rhizobium phage vB_RleM_P10VF | NC_025429 | 1.0450 | 0.0007 | TRUE |
| Vibrio phage SHOU24 | NC_023569 | 1.0127 | 0.0019 | TRUE |
| Acinetobacter phage IME_AB3 | NC_023590 | 1.0035 | 0.0053 | TRUE |
| Rhodococcus phage ReqiDocB7 | NC_023706 | 1.0299 | 0.0010 | TRUE |
| Microbacterium phage vB_MoxS-ISF9 | NC_023859 | 1.0005 | 0.0086 | TRUE |
| Shewanella sp. phage 1/40 | NC_025470 | 1.0239 | 0.0012 | TRUE |
| Shewanella sp. phage 1/4 | NC_025436 | 1.0260 | 0.0011 | TRUE |
| Acinetobacter phage vB_AbaM_Acibel004 | NC_025462 | 1.0061 | 0.0037 | TRUE |
| Pseudomonas phage phiPto-bp6g | NC_023718 | 1.0142 | 0.0018 | TRUE |
| Vibrio phage phi-A318 | NC_025822 | 1.0263 | 0.0011 | TRUE |
| Anabaena phage A-4L | NC_024358 | 1.0279 | 0.0011 | TRUE |

Table S2: The comparison of assembly precision using filtered reads and total reads for 20% viral reads and 80% host reads.

|  | |  |  |  |  |  |  |  |
| --- | --- | --- | --- | --- | --- | --- | --- | --- |
| simulation | 20M | | | | 40M | | | |
|  | Filtered reads | | Total reads | | Filtered reads | | Total reads | |
| contig length | contig_num | Precision | contig_num | Precision | contig_num | Precision | contig_num | Precision |
| 300 | 12544 | 0.947 | 12458 | 0.911 | 12115 | 0.953 | 9985 | 0.928 |
| 500 | 6812 | 0.935 | 7464 | 0.883 | 6902 | 0.934 | 6746 | 0.893 |
| 1000 | 4473 | 0.922 | 4261 | 0.861 | 4963 | 0.908 | 4649 | 0.868 |
| 2000 | 1575 | 0.943 | 1342 | 0.876 | 1672 | 0.903 | 1506 | 0.828 |
| 3000 | 1196 | 0.926 | 949 | 0.872 | 1242 | 0.915 | 1134 | 0.794 |
| 5000 | 835 | 0.941 | 623 | 0.876 | 844 | 0.921 | 642 | 0.814 |
| 10000 | 469 | 0.925 | 476 | 0.722 | 642 | 0.862 | 565 | 0.643 |
| assembly length (Mb) | 36.322 |  | 33.801 |  | 41.230 |  | 36.003 |  |
|  |  |  |  |  |  |  |  |  |
